# Effect of Trap Behavior on the Reliability Instability of Metamorphic Buffer in InAlAs/InGaAs MHEMT on GaAs

**DOI:** 10.3390/ma16186138

**Published:** 2023-09-09

**Authors:** Ki-Yong Shin, Ju-Won Shin, Walid Amir, Surajit Chakraborty, Jae-Phil Shim, Sang-Tae Lee, Hyunchul Jang, Chan-Soo Shin, Hyuk-Min Kwon, Tae-Woo Kim

**Affiliations:** 1 Department of Electrical, Electronic and Computer Engineering, University of Ulsan, Ulsan 44610, Republic of Korea; 2 Korea Advanced Nano Fab Center, Suwon-si 16229, Republic of Korea; 3 Department of Semiconductor Processing Equipment, Semiconductor Convergence Campus of Korea Polytechnics, Anseong-si 17550, Republic of Korea

**Keywords:** reliability, traps, MHEMT, fast transient, pulsed I–V, 1/*f* (low-frequency) noise

## Abstract

Our investigation focused on assessing the influence of the metamorphic buffer in metamorphic high-electron-mobility transistors (MHEMT) that were grown on GaAs substrates. While an MHEMT exhibited elevated off-state current levels, its direct current (DC) and radio frequency (RF) traits were found to be comparable to those of InP-based lattice-matched high-electron-mobility transistors (LM-HEMTs). However, the Pulsed I–V measurement results confirmed the presence of the fast transient charging effect, leading to a more substantial degradation in drain current observed in MHEMT. In addition, through the low-frequency noise characteristics, it was confirmed that the dominant trapping location was located in the bulk site. The slope of the 1/*f* noise measurement indicated that the primary trapping site was in proximity to the bulk traps. The carrier-number-fluctuation (CNF) model was employed to extract the bulk trap density (*N_t_*). For the LM-HEMTs, the value was at 3.27 × 10^16^ eV^−1^·cm^−3^, while for the MHEMT, it was 3.56 × 10^17^ eV^−1^·cm^−3^.

## 1. Introduction

The demand for transistors operating at frequencies exceeding 300 GHz is witnessing a notable surge across an array of sectors, encompassing next-generation communication, medical imaging systems, Internet of Things (IoT) applications, radar technologies, and satellite communication systems. Notably, high-electron-mobility transistors (HEMTs) featuring InGaAs channels have garnered considerable attention due to their remarkable properties, such as high electron mobility (*μ_n_eff_*) and virtual source injection velocity (*v_inj_*) [1]. This renders them exceptionally suitable for driving forward the realm of terahertz communication and serving as proficient low-noise amplifiers. The unparalleled high-electron-mobility properties of these devices have led to pseudomorphic high-electron-mobility transistors (pHEMTs) on InP substrates, achieving remarkable milestones, boasting cut-off frequencies (*f_T_*) and maximum oscillation frequencies (*f_max_*) surpassing the 700 GHz threshold. This achievement of reaching among the highest values for field effect transistors owes its success to the inherently elevated high electron mobility characteristics [2,3,4].

While InGaAs HEMTs have undeniably showcased impressive performance benchmarks on InP substrates, these substrates have their own limitations. Their relatively diminutive size, often measuring 3 to 4 inches, carries certain economic disadvantages owing to the exorbitant production costs associated with such dimensions. Additionally, their inherent fragility compounds these limitations. Furthermore, the process technology aligned with these smaller-sized wafers remains incomplete, translating to lower yields. In a bid to surmount these challenges, researchers have diligently worked on the development of metamorphic HEMTs (MHEMT) on GaAs substrates. The rationale behind this lies in the manifold advantages presented by GaAs substrates, such as their cost effectiveness facilitated by large-volume manufacturing, augmented mechanical robustness, and the potential for scaling the substrate size to 6 inches.

Various studies have been conducted on MHEMT, which offers numerous advantages in substrates. These numerous studies have resulted in MHEMTs exhibiting DC and RF performance equivalent to the pHEMTs achieved through various buffer structures [5,6,7]. Since both devices have shown high RF performance, they were expected to find applications in various fields requiring such capabilities. Among these, especially for commercial applications like low-noise and high-power millimeter-wave amplifiers, the reliability performance of pHEMTs and MHEMTs has been a significant concern. The degradation mechanisms in pHEMTs and MHEMT on InP and GaAs substrates have been studied, taking into account factors such as the diffusion of the gate metals [8,9,10], fluorine contamination [11], hydrogen absorption into Ti metallization [8,12], corrosion associated with Al oxidation [10], gate sinking [13,14], and ohmic contact degradation [13,15]. These studies have mainly discussed the impact of changes in the process. Apart from this, research has predominantly centered on degradation mechanism analysis, including device lifetime and property degradation due to stress. Additionally, representative studies have been conducted on high-temperature operating-lifetime tests that cause a rapid decrease in transconductance, hot carrier-induced impact ionization at the gate, or bias stressing [8,14,15,16,17,18,19,20,21,22,23,24,25]. During this research, factors related to lifetime and bias stress evaluation become crucial in assessing the reliability performance, especially when considering the operating environment of actual devices. However, it is essential to note that a significant portion of the research, excluding this, has primarily concentrated on reliability evaluation within various fabrication processes, without a specific focus on differences in device structure or substrate.

The integration of a metamorphic buffer layer into the device structure, strategically positioned between the substrate and the HEMT layer, inevitably introduces a lattice mismatch factor with the substrate. This, in turn, culminates in a marked elevation in dislocation density, further compounded by the manifestation of a rough surface morphology [26]. As a direct consequence of this lattice mismatch, notwithstanding the inherent advantages offered by the substrate, a notable degree of instability in terms of device reliability surfaces. Consequently, a deeper and more comprehensive understanding of the intricate dynamics responsible for this reliability instability assumes a paramount role. It becomes imperative to unravel the underlying factors triggering this phenomenon while concurrently discerning the intricate interplay between trap sites and dislocation.

In this work, we investigated the reliability instability associated with the lattice mismatched metamorphic buffer in MHEMT on GaAs substrates, and the instability was compared with lattice-matched HEMTs (LM-HEMTs) on InP substrates having the same structure identical to that of the MHEMT. Both types of devices were subjected to the same fabrication process for the purpose of comparison. In both MHEMT and LM-HEMTs, which demonstrated similar DC and RF characteristics, we employed pulsed I–V measurement to investigate the change in drain current resulting from the fast transient charging effect due to the structural differences. Additionally, 1/*f* noise measurements were conducted to identify the dominant trap sites in the devices. For quantitative comparison, the process of extracting the bulk trap density of the device was performed using DC and 1/*f* noise characteristics.

## 2. Experimental Details

**Figure 1:**
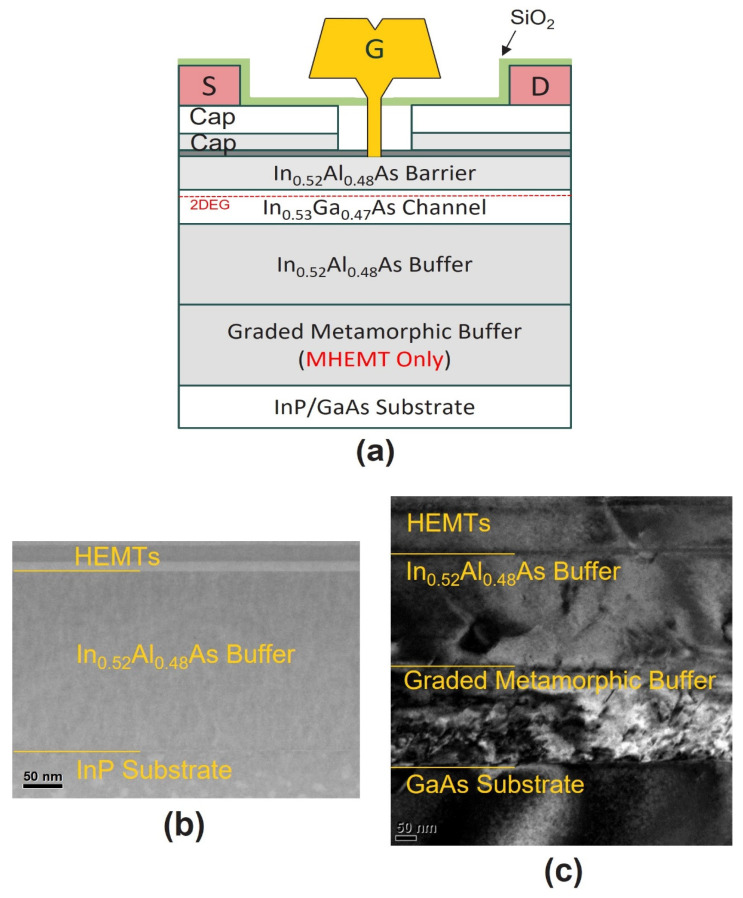
(**a**) This schematic depicts the cross-section view of HEMTs grown on two different substrates. Additionally, TEM images of the cross-section are provided, showing (**b**) LM-HEMTs and (**c**) MHEMT.

LH-HEMTs were grown on InP (3-inch), and MHEMT were grown on larger GaAs (6-inch) substrates through molecular beam epitaxy (MBE). The epitaxial layer, arranged from top to bottom, consisted of the following components: a heavily doped 40 nm multilayer cap (comprising In_0.7_Ga_0.3_As, In_0.53_Ga_0.47_As, and In_0.52_Al_0.48_As), a 4 nm InP etch stopper, an 8 nm In_0.52_Al_0.48_As barrier, Si δ-doping, a 3 nm In_0.52_Al_0.48_As spacer, a 10 nm In_0.53_Ga_0.47_As channel, and a 100 nm In_0.52_Al_0.48_As buffer. The N^+^ type 10 nm In_0.7_Ga_0.3_As and 20 nm In_0.53_Ga_0.47_As capping layer was heavily doped with 5.00 × 10^19/^cm^3^. Also, the N^+^-type 10 nm In_0.52_As_0.48_As capping layer was doped with a doping density of 3.00 × 10^19^/cm^3^. And the Si δ-doping density between the InAlAs barrier and spacer was 5.0 × 10^12^/cm^2^ for both epitaxies. In LM-HEMTs epitaxy, the measured sheet carrier density (*N_S_*) of the 2-dimensional electron gas (2DEG) in the InGaAs channel layer was 2.9 × 10^12^/cm^2^, and the electron hall mobility (*μ_n_*) was 10,060 cm^2^/V·s. The measured MHEMT *N_S_* and *μ_n_* were 3.0 × 10^12^/cm^2^ and 8950 cm^2^/V·s, respectively. To check *N_S_* and *μ_n_*, van der Pauw Hall measurements were carried out. This was measured after etching the multi-capping layer. The epitaxy used in the experiment was a commercial epitaxy. InP substrates were utilized to form LM-HEMTs with this structure, whereas GaAs substrates were employed for growing MEHMT with a metamorphic buffer (graded). Figure 1a illustrates a schematic of the cross-sectional view of the InAlAs/InGaAs HEMTs. Transmission electron microscopy (TEM) was used to confirm the discrepancies in dislocation and rough surface morphology between LM-HEMTs and MHEMT. Figure 1b presents TEM images of LM-HEMTs, exhibiting a clean interface between the substrate and the In_0.52_Al_0.48_As buffer. MHEMT TEM images in Figure 1c confirm the existence of a significant dislocation density and rugged surface generated from the mismatch of lattice constants between the substrate and the metamorphic buffer. Figure 1c tries to clearly confirm the dislocation using a bright field TEM image. The purpose of this selection was to enhance the clarity in order to emphasize the discrepancies in dislocation between LM-HEMTs and MHEMT to make the observed variances more apparent and distinguishable.

The fabrication process for both MHEMT and LM-HEMTs included mesa isolation through phosphoric-acid-based wet chemical etching for device isolation. Additionally, non-alloyed Ohmic contacts were established using a Molybdenum (Mo, 10 nm)/Titanium (Ti, 10 nm)/Platinum (Pt, 30 nm)/Gold (Au, 350 nm)/Nickel (Ni, 5 nm) stack for the source and drain electrode formation. At this time, the Ni metal located at the top of the Ohmic metal stack was used to improve the adhesion between SiO_2_ and the ohmic metal. SiO_2_ passivation was achieved through sputtering. This improves adhesion during photoresist coating to form a T-gate, increases the stability of the T-gate structure, and helps to reduce the side etch length in the recess etch process before gate deposition. And then, sub-200 nm T-gates were formed using electron beam lithography. The SiO_2_ passivation layer, which was applied to the active area, underwent reactive ion etching before the gate was deposited. Then, a multilayer cap with significant doping was subjected to etching through the utilization of an etchant based on phosphoric acid. Following this process, the gate was formed, consisting of the Platinum (Pt, 8 nm)/Titanium (Ti, 20 nm)/Platinum (Pt, 20 nm)/Gold (Au, 350 nm) stack. Subsequently, a Pt sinking process was conducted at 250 °C to bury Pt on the barrier layer. This contact serves as a fundamental element in mitigating the gate leakage current of the fabricated devices. The fabricated devices featured specific dimensions, including a gate width (*W_g_*) of 50 μm, a source-to-gate distance (*L_SG_*) of 1 μm, and a drain-to-source distance (*L_DG_*) of 1 μm.

## 3. Results and Discussion

### 3.1. Devices Characteristics

**Figure 2:**
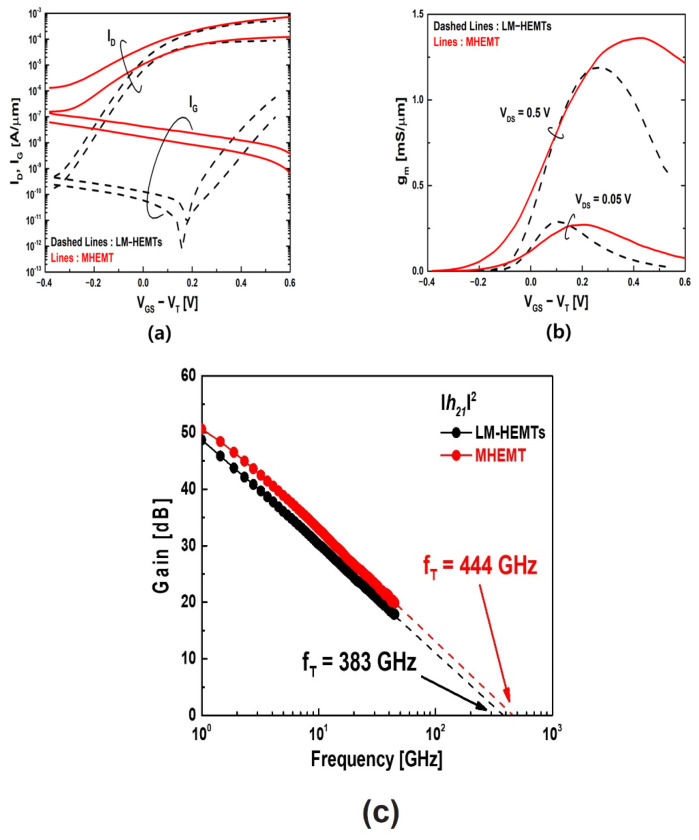
The characteristics with a gate length (*L_g_*) of 50 nm are as follows: (**a**) *I_D_* and *I_G_* characteristics at *V_DS_* = 0.05 V and 0.5 V. (**b**) *g_m_* characteristics at *V_DS_* = 0.05 V and 0.5 V. (**c**) RF performance at *V_DS_* = 0.8 V, peak *g_m_*.

Before conducting the reliability analysis, we assessed the characteristics of the fabricated devices. Figure 2a displays the *I_D_* and *I_G_* performance of devices with a gate length (*L_g_*) of 50 nm at *V_DS_* values of 0.05 V and 0.5 V. Due to a substantial gate leakage current, MHEMT on GaAs substrates exhibited elevated off-state currents, resulting in a higher subthreshold swing (SS) of 143 mV/decade, while for LM-HEMTs, it was only 66 mV/decade. And the drain-induced barrier lowering (DIBL) values were 79 mV/V for LM-HEMTs and 298 mV/V for MHEMT. In Figure 2b, at a *V_DS_* of 0.5 V, the MHEMT had a maximum *g_m_* value of 1.35 mS/μm, while LM-HEMTs had a 1.19 mS/μm. The contact resistance (R_C_) and sheet resistance (R_sh_) of the two devices were measured through the Transmission Line Method (TLM) pattern. This R_sh_ is related to the In_0.7_Ga_0.3_As, In_0.53_Ga_0.47_As, and In_0.52_Al_0.48_As multi-capping layer. The R_sh_ of LM-HEMTs is 42.40 Ω/□, and the R_C_ is 12.80 Ω μm. MHEMT showed R_sh_ and R_C_ of 41.63 Ω/□ and 14.16 Ω μm. In addition, LM-HEMTs and MHEMT recorded resistances (R_ON_) of 476 Ω μm and 418 Ω μm, respectively. Figure 2c presents the obtained short-circuit current gain (|h_21_|^2^), featuring a gate length (*L_g_*) of 50 nm at a *V_DS_* of 0.8 V and in close proximity to the conditions where the *g_m_* reaches its maximum value. The entire RF characterization spanned from 1 to 45 GHz and was executed using a network analyzer, with the calibration being performed off the wafer. The de-embedding process of the S-parameters’ data was carried out using wafers’ open and short patterns, which helped to remove the parasitic pad components. Through extrapolation, *f_T_* was obtained to be 383 GHz and 444 GHz for LM-HEMTs and MHEMT, respectively. And *f_max_* values recorded in similar bias conditions were 450 GHz and 427 GHz for LM-HEMTs and MHEMT. As such, high off-state current and high DIBL and swing values are the main concerns of MHEMT. But despite the lattice mismatch between the graded metamorphic buffer and GaAs, as well as the high off-state current, their DC and RF performance were comparable with those of LM-HEMTs. Higher *g_m_* and *f_T_* values were recorded for MHEMT, which implied that phosphoric-acid-based wet etching of the gate recess resulted in different etch depths for LM-HEMTs and MHEMT. Although the buffer leakage current at 1 V was 23 and 80 nA for LM-HEMTs and an MHEMT, respectively, the difference in gate leakage current was greater by three orders of magnitude. This implied that the buffer structure strongly influenced the overall structure and properties.

### 3.2. Fast Transient Charging Effect with Pulse I–V

To validate the presence of the fast transient charging effect in the two types of devices, we utilized the pulsed I–V measurement and single-pulse *I_D_*–*V_G_* techniques. These measurements were conducted using a semiconductor parameter analyzer in conjunction with the waveform generator module [27,28,29,30,31,32]. During the pulsed I–V measurements, a single short pulse was applied to the gate and drain, and the resulting *I_D_* was measured. When a signal is applied, the carriers of the channel are charged to the defect of the barrier. The migrated electrons cause rapid *V_t_* and *I_D_* degradation. This charge-trapping effect follows two steps. The first charging process at the shallow trap site near the InAlAs barrier’s conduction band is expected to have a short charging time because the trap energy is very low, and the density of states is high in the channel’s conduction band. Next, slow transient charging can be attributed to the capture of secondary electrons, which is induced from the charges trapped due to the fast charging process. These are called deep traps and are located deeper in the InAlAs conduction band than shallow traps.

**Figure 3:**
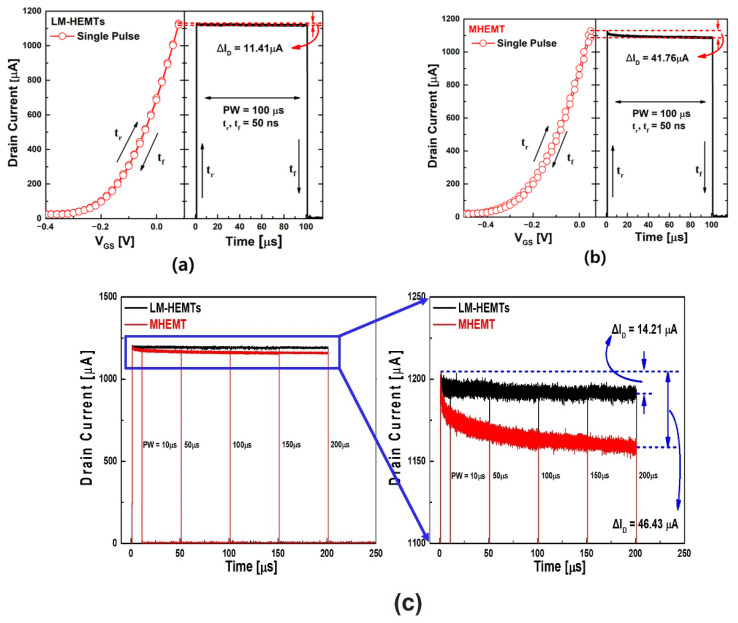
Single-pulse *I_D_V_G_* measurement and rapid drain current degradation as a function of time corresponding to a pulsed *I_D_*–*V_G_* sweep for (**a**) LM-HEMTs and (**b**) MHEMT. (**c**) *I_D_*–time sweeps for both types of samples as a function of the PW (10, 50, 100, 150, 200 μs), with *t_r_* and *t_f_* maintained constant (50 ns).

To effectively minimize the fast transient charge-trapping effect when applying the trapezoidal *V_G_* pulse, rise (*t_r_*) and fall (*t_f_*) times of 50 ns were utilized, which were very short. If *t_r_* and *t_f_* are not minimized, charge trapping continuously occurs during the time the signal is applied, just like non-pulse signals. At this time, the current characteristics after the charge-trapping phenomenon has already occurred are checked. The fast transient charge-trapping effect is known to occur when a pulse shorter than 1 ms is applied [27]. Figure 3a,b display the results of single-pulse measurements for the two types of devices, presented in either the pulse–voltage or pulse–time domain. Indeed, charge trapping had an impact on *V_t_*, as evidenced by the hysteresis observed in the *I_D_*–*V_G_* curve and the degradation in the drain current (*I_D_*) over time in the *I_D_*–time domains. Notably, these changes were more pronounced in MHEMT. The threshold voltage shift (Δ*V_t_*) was calculated using the equation [27]

(1) $$\Delta V_{t}=\frac{\Delta I_{D}\cdot (V_{G}-V_{t})}{I_{D}}$$

The decrease in drain current (Δ*I_D_*) during the pulse width (PW) is calculated as the difference between the maximum *I_D_* before the PW and the drain current during the PW. *V_G_* represents the pulse amplitude. Equation (1) is applicable when the decrease in mobility because of the high vertical field of the channel is not large owing to the high gate bias.

In Figure 3c, the fast transient charging effect was confirmed under various PW conditions (10, 50, 100, 150, 200 μs) with *t_r_* and *t_f_* being maintained constant. The degradation of the drain current at different PW was attributed to the charge-trapping effect. Specifically, for PW = 200 μs, the drain current degradation in LM-HEMTs and MHEMT was observed to be 14.21 μA and 46.43 μA, respectively. This suggests that MHEMT experienced a higher degradation in the drain current compared to LM-HEMTs under the same pulse-width conditions. Notably, the latter value is more than triple the former value. The higher degradation of the drain current in MHEMT compared to LM-HEMTs at the same pulse width suggests that channel carriers were more prone to getting trapped within the defective region of the device during the pulsed measurement. Even when the PW is continuously increased up to 200 μs, in the case of LM-HEMTs, the drain current value saturates after around 50 μs and does not significantly decrease. On the other hand, MHEMT shows a phenomenon in which the current continuously decreases until a PW of 200 μs is reached. It can be said that deep inside the semiconductor, many traps are continuously created. This trapping phenomenon leads to a more significant reduction in drain current in MHEMT, highlighting the impact of the fast transient charging effect on their performance.

### 3.3. 1/f (Low-Frequency) Noise

**Figure 4:**
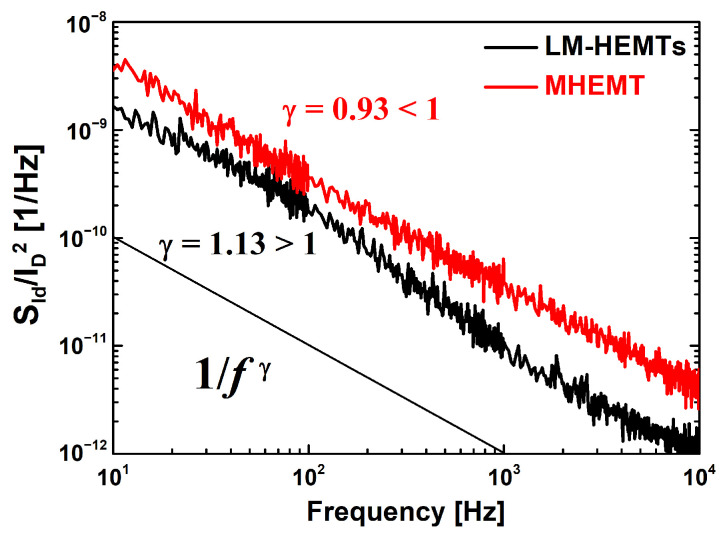
Comparison of the normalized drain current noise spectral density (*S_Id_/I_D_*^2^) at *V_DS_* = 0.05 V and *I_D_* = 1 A/µm.

To gain insights into the primary locations of the trapping phenomena resulting from the defect site in response to the fluctuation of the channel carriers, 1/*f* noise measurements were conducted [32]. This is an effective measurement method for analyzing interfaces that can cause device performance degradation. This measurement was performed using a low-noise current amplifier in conjunction with a signal analyzer. Frequencies up to 10^4^ Hz were covered at *V_DS_* = 0.05 V. Figure 4 shows the normalized drain current noise (*S_Id_/I_D_*^2^) for *I_D_* = 1 A/µm. A lower 1/*f* noise characteristic indicates fewer traps or defects. LM-HEMTs had a lower drain current noise density than MHEMT. The frequency dependence of *S_Id_*/*I_D_^2^*, also known as flicker noise or 1/*f* noise, can be described using a power-law equation of 1/*f* ^γ^. In the given frequency range, the noise data were fitted using the 1/*f*^γ^ function, and the frequency exponent (γ) was determined. The value of γ provides crucial information about the nature of the trapping mechanisms and their influence on the noise characteristics in the devices [33,34]. The frequency exponent being close to 1 in 1/*f* noise measurements typically suggests that the dominant traps responsible for the noise are nearly constant in terms of energy and depth [28]. This behavior indicates that these traps have a relatively uniform impact on the noise characteristics across different frequencies, leading to the observed power-law behavior in the 1/*f* noise spectrum. [33,34]. A value of γ less than 1 for the MHEMT suggests that the primary trapping site is situated near the bulk traps, which are positioned away from the channel interface. On the other hand, the LM-HEMTs with a γ greater than 1 indicate that the location of the dominant trapping phenomena is situated at the interface of the InGaAs channel. The difference in γ values provides valuable insights into the nature and locations of the dominant traps contributing to the 1/*f* noise in each type of device.

There are several physical theories that provide explanations of 1/*f* noise [35,36]. According to the McWhorter model, the initial source of 1/*f* is the channel’s fluctuating carrier number as a result of carrier trapping and de-trapping. On the other hand, the Hooge model suggests that noise results from carriers’ fluctuating mobility caused by phonon-scattered carriers. In the case of the device used in this study, it was determined that the device’s noise characteristics followed the McWhorter model. So, to achieve a quantitative comparison, we derived *N_t_* utilizing the carrier-number-fluctuation (CNF) model [31,37,38,39]:

(2) $$\frac{S_{Id}}{I_{D}^{2}}={\left(\frac{g_{m}}{I_{D}}\right)}^{2}S_{Vfb}$$

(3) $$\mathrm{with}\;S_{Vfb}=\frac{q^{2}N_{t}kT\lambda }{WLC_{ins}^{2}f}$$

**Figure 5:**
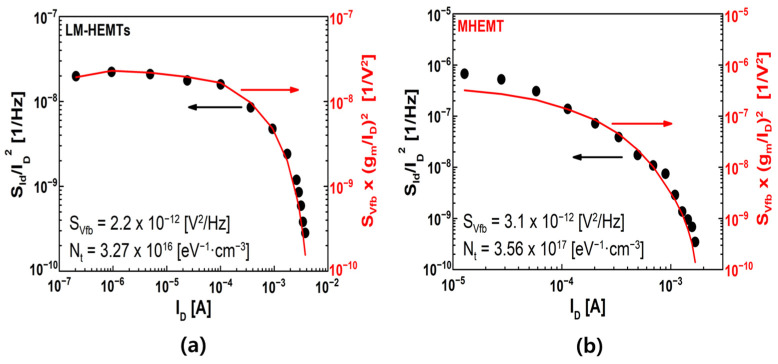
Illustrate the *S_Id_*/*I_D_*^2^ represented by black dots and the (*g_m_*/*I_D_*)^2^ shown as red curves for (**a**) LM-HEMTs and (**b**) MHEMT. These measurements were conducted at a *V_DS_* of 0.05 V and *L_g_* of 50 nm.

*S_Vfb_* represents the flat band power spectral density, *kT* stands for the thermal energy, *WL* corresponds to the channel area, *C_ins_* signifies the insulator capacitance, *f* represents the frequency, and *N_t_* denotes the bulk trap density. In addition, *λ* is the tunneling attenuation distance and can be calculated using the formula *λ* = [4π(2*m* ∗ *Φ_B_*)^1/2^/*h*]^−1^*,* where *Φ_B_* stands for the barrier height and m* denotes the effective mass. In the CNF model, both the terms *S_Id_*/*I_D_*^2^ and (*g_m_*/*I_D_*)^2^ exhibit similar variations across a range of drain currents or gate voltages. As depicted in Figure 5a,b, both *S_Id_*/*I_D_*^2^ and (*g_m_*/*I_D_*)^2^ display comparable variations over multiple decades as the drain current changes. These measurements were conducted at 10 Hz and for devices with an *L_g_* of 50 nm. The determined values of *S_Vfb_* were 2.2 × 10^−12^ and 3.1 × 10^−12^ V^2^·Hz^−1^ for LM-HEMTs and MHEMT, respectively. Correspondingly, the calculated *N_t_* values were 3.27 × 10^16^ eV^−1^·cm^−3^ for LM-HEMTs, while for the MHEMT, it was 3.56 × 10^17^ eV^−1^·cm^−3^. MHEMT has a trap density one order higher than LM-HEMTs.

## 4. Conclusions

In conclusion, this study investigated the reliability instability associated with the lattice-mismatched metamorphic buffer in InAlAs/InGaAs MHEMT and compared it with LM-HEMTs. Both types of devices underwent the same fabrication process to enable a direct comparison. MHEMT exhibited poor DIBL characteristics with higher off-state current and larger swing values due to high gate leakage current, but their DC and RF performances were found to be comparable to LM-HEMTs. Pulsed I–V measurements revealed the presence of the fast transient charging effect, leading to more than threefold greater degradation of the drain current in MHEMT compared to LM-HEMTs. In addition, it was shown that the drain current decrease over time but does not saturate and continues to decrease due to charge trapping. Furthermore, 1/*f* noise measurements provided valuable insights into the dominant trap sites in the devices. The results showed that the dominant trapping location in MHEMT was situated near the bulk traps, whereas for LM-HEMTs, it was at the InGaAs channel interface. The *N_t_* value obtained using the CNF model was 3.27 × 10^16^ eV^−1^·cm^−3^ for LM-HEMTs, whereas was is an order of magnitude higher, 3.56 × 10^17^ eV^−1^·cm^−3^, for MHEMT.

The presence of a lattice-mismatched metamorphic buffer led to a dislocation density and rough surface, which contributed to reliability instability in MHEMT. Understanding the mechanisms behind this instability is crucial for future research aiming to improve the reliability of the metamorphic buffer layer, enhance the quality of each layer in order to create a better epitaxy, and optimize device performance.

This study experimentally determined the degradation mechanism caused by the graded metamorphic buffer layer, shedding light on the structural instability of MHEMT. The findings suggest the need for further exploration to enhance the reliability and stability of the metamorphic buffer layer and mitigate its adverse effects in future device designs.

## Data Availability

The data presented in this study are available on request from the corresponding author.
